# Complex Relationship Between Critical Flicker Fusion Frequency and Established Cognitive Tests Unveiled by Hyperbaric Exposure

**DOI:** 10.3390/biology15030242

**Published:** 2026-01-28

**Authors:** Natalia D. Mankowska, Rita I. Sharma, Anna B. Marcinkowska, Pawel J. Winklewski, Jacek Kot

**Affiliations:** 1 Applied Cognitive Neuroscience Lab, Department of Neurophysiology, Neuropsychology and Neuroinformatics, Medical University of Gdansk, 80-210 Gdansk, Poland; anna.marcinkowska@gumed.edu.pl; 2 Department of Neurophysiology, Neuropsychology and Neuroinformatics, Medical University of Gdansk, 80-210 Gdansk, Poland; r.sharma@gumed.edu.pl (R.I.S.); pawel.winklewski@gumed.edu.pl (P.J.W.); 3 2nd Department of Radiology, Medical University of Gdansk, 80-210 Gdansk, Poland; 4 National Centre for Hyperbaric Medicine, Institute of Maritime and Tropical Medicine in Gdynia, Medical University of Gdansk, 81-519 Gdynia, Poland; jkot@gumed.edu.pl

**Keywords:** critical flicker fusion frequency, hyperbaric exposure, cognitive performance, air, heliox, trimix, nitrogen effects, arousal

## Abstract

Critical flicker fusion frequency (CFFF) has been proposed as a rapid marker of central nervous system arousal state, but its relationship to cognitive performance under hyperbaric stress remains unclear. Forty healthy adults (20 women; age 19–46 years) underwent three hyperbaric exposures at 4 ATA (equivalent to 30 m seawater depth) while breathing air, heliox, and trimix in randomized order. CFFF and cognitive performance (Simon task, Digit Span, Corsi Block-Tapping) were assessed before compression, at 4 ATA, and after decompression. Both CFFF and reaction times increased modestly at 4 ATA across all breathing gases (3–5% elevation, *p* < 0.05). Following decompression, however, these measures showed divergent recovery patterns: CFFF normalized completely after heliox and trimix, but remained partially elevated after air breathing. In contrast, reaction times improved substantially after decompression (10–15% faster than baseline) regardless of breathing gas, reflecting practice effects. Weak correlations emerged between specific CFFF components and executive cognitive measures, while memory performance remained stable throughout all conditions. CFFF and cognitive performance exhibit some parallel increases under hyperbaric stress but most likely capture largely independent aspects of neural function. Breathing gas composition selectively influences CFFF recovery dynamics, with nitrogen producing prolonged neural effects relevant for assessing post-dive cognitive readiness.

## Simple Summary

Divers and people working in pressurized environments often experience changes in their ability to think clearly and react quickly. To ensure safety, we need simple tools that can quickly assess whether someone’s brain is functioning normally under these challenging conditions. This study examined whether a rapid visual test called the “flicker test”, which measures how fast a person can detect changes in flickering light, could serve as a practical indicator of cognitive performance when people breathe different gas mixtures in a pressure chamber simulating a 30 m dive. We tested 40 healthy adults three times, each time with a different breathing gas: atmospheric air, a helium–oxygen mixture, and a helium–oxygen–nitrogen mixture. We found that pressure increased flicker test scores regardless of the gas breathed, but only while breathing air did the scores remain elevated after returning to normal pressure. The flicker test results showed weak correlations with cognitive performance tests, suggesting they measure somewhat different aspects of brain function. These findings indicate that people breathing air may need more recovery time before resuming demanding cognitive tasks after diving, while nitrogen-free gases allow faster return to baseline brain function, which has important implications for dive planning and post-dive work protocols.

## 1. Introduction

The flicker test, which assesses critical flicker fusion frequency (CFFF), has gained attention as a rapid, non-invasive tool for evaluating central nervous system arousal state. CFFF measures the frequency at which a flickering light source ceases to be perceived as flickering and instead appears continuous. This perceptual threshold reflects the temporal resolution capacity of the visual system and has been consistently linked with levels of cortical arousal and information processing efficiency [1,2,3,4,5,6]. A key advantage of CFFF as a measurement tool is its resistance to learning effects and its capacity to capture instantaneous arousal states [4,7,8], making it valuable for assessing cognitive readiness in extreme or challenging environments, where rapid evaluation is essential and repeated measurements over short intervals are often required [1,4].

Hyperbaric environments present a unique physiological challenge characterized by increased ambient pressure, altered partial pressures of respiratory gases, and potential exposure to inert gas narcosis. Under such conditions, CFFF has been shown to change in a dose-dependent manner, suggesting modulated neuronal excitability in response to hyperbaric stress [9,10]. This elevation in CFFF is particularly evident at moderate depths—for example, at 4 ATA (equivalent to 30 m of seawater)—where multiple factors including hyperoxia, increased inert gases partial pressure, and mechanical effects of pressure converge to modulate central nervous system function.

The choice of breathing gas mixture fundamentally alters hyperbaric exposure. Air, containing 78% nitrogen, exposes divers to substantial nitrogen narcosis risk. In contrast, helium-based mixtures (heliox, trimix) reduce or eliminate nitrogen, potentially minimizing narcotic effects due to helium’s low lipid solubility and different physicochemical properties [11,12]. However, systematic comparisons of CFFF responses across different breathing gases under controlled conditions remain limited.

The relationship between CFFF and cognitive performance under hyperbaric conditions is also poorly understood. Under normobaric conditions, CFFF shows weak or absent correlations with neuropsychological test performance [7,13,14,15], suggesting these measures capture largely independent aspects of neural function. However, this relationship may strengthen under physiological stress, where arousal state becomes rate-limiting for cognitive performance. Theoretical frameworks suggest that arousal-regulating systems, particularly the locus coeruleus-noradrenergic (LC–NE) system [16,17,18], may differentially modulate prefrontal-dependent executive functions [19,20,21] versus basic memory processes [22,23], potentially creating domain-specific CFFF-cognition relationships under challenging conditions.

The present study addresses these gaps by examining CFFF and cognitive performance during hyperbaric exposure at 4 ATA (30 m) while breathing air, heliox, or trimix. Accordingly, we proposed the following hypotheses:

1. CFFF changes will correlate with Simon task but not with Digit Span or Corsi Block-Tapping performance,
2. Different breathing gas mixtures will produce differential effects on CFFF. Specifically, helium-rich mixtures should show less pronounced changes than nitrogen-rich air, due to helium’s minimal narcotic properties and rapid tissue kinetics,
3. Changes occurring at 4 ATA will not persist after returning to normobaric conditions for helium-rich gases but may show prolonged elevation for nitrogen-rich breathing mixtures.

An important secondary aim was exploratory: to characterize the time course of CFFF changes following decompression, particularly whether different breathing gas compositions would produce distinct recovery trajectories. By systematically varying breathing gas composition while controlling exposure depth and duration, this study aims to clarify the neural and cognitive effects of hyperbaric environments and inform safety practices in recreational and professional diving.

## 2. Materials and Methods

The study was conducted in the Department of Hyperbaric Medicine and Maritime Rescue—National Centre for Hyperbaric Medicine in Gdynia, which is part of the Medical University of Gdansk (Poland). Experimental procedures were conducted in accordance with the Declaration of Helsinki and were approved by the Bioethics Committee for Scientific Research at Medical University of Gdansk (242/2020). Inclusion criteria comprised: age between 18 and 55 years and good general health. Exclusion criteria were: a history of cardiovascular diseases not under strict medical supervision, history of thoracic or otolaryngologic surgery, pulmonary diseases, and pregnancy. Also, participants were excluded if they reported: (1) daily use of any psychoactive substance except caffeine; (2) alcohol consumption exceeding 14 standard units per week for men or 7 for women (low-risk drinking thresholds); (3) current use of prescription psychoactive medications (benzodiazepines, stimulants, antidepressants, antipsychotics); (4) any recreational drug use in the 30 days prior to participation. Participants self-reported substance use during screening and were instructed to abstain from alcohol (24 h) and caffeine (2 h) before each testing session. Prior experience with hyperbaric chambers or wet diving was not required for participation.

### 2.1. Participants

A total of 71 individuals (32 men and 39 women) were initially recruited for the study. Of these, 25 were excluded during screening (failure to meet inclusion criteria or declined participation), leaving 46 qualified participants. During the study, six participants withdrew from further participation, and their data were excluded from subsequent analyses. The final sample consisted of 40 participants (20 women), aged 19 to 46 years (M = 31.75, SD = 8.54).

### 2.2. Procedure

Each participant was invited to attend three separate sessions at the National Centre for Hyperbaric Medicine in Gdynia (Poland). During each visit, participants completed a battery of tests at three distinct time points:

- Stage “before”: prior to entering the chamber (1 ATA, ambient air),
- Stage “4 ATA”: during exposure at 4 ATA while breathing one of the experimental gas mixtures,
- Stage “after”: following decompression back to 1 ATA (ambient air).

A different breathing gas mixture was administered during each of the three sessions, with the participant inhaling it at 4 ATA. The following mixtures were used: air (20.8% oxygen, 78% nitrogen), heliox (21% oxygen, 79% helium), and trimix (21.6% oxygen, 36.7% nitrogen, 41.7% helium). In total, 40 subjects completed the experiment with air (20 women), 36 with heliox (17 women), and 37 with trimix (18 women). The number of participants differed across individual sessions, as some subjects withdrew from further participation in the study without providing a reason.

Each session was separated by a mandatory 24 h break. The order of breathing gas mixtures was partially randomized for each participant. For the first two sessions, participants selected their preferred date from two predetermined options; breathing gas assignment for these dates was randomly determined using computer-generated sequences. The third session used the remaining gas and was scheduled by participant availability.

Participants were not informed about the specific breathing gas mixture used during each session to maintain blinding. Only the chamber operator and attendant were aware of the composition. Details regarding the gas mixture were disclosed to participants upon request, but only after the testing procedures had been completed.

All dives followed identical compression and decompression profiles regardless of breathing gas mixture. The participant’s compression rate during each session was approximately 5–6 msw/min to 30 msw and was adjusted to their ability and experience in pressure equalization. Exposure at 4 ATA lasted 25–30 min, followed by decompression with two safety stops, based on official safety tables (Polish Ministry of Health, 2007, as described by Kot & Sićko [24]). No decompression stops were required for these exposures. The identical depth-time profiles across conditions allow us to attribute observed differences in recovery patterns to gas composition rather than differential decompression stress. No emergency procedures were required across 59 completed sessions. Due to increasing pressure in the chamber during each exposure, the temperature in the hyperbaric chamber oscillated between 31 and 37 degrees Celsius (M = 34.53, SD = 1.49) and did not differ significantly between trials with different gas mixtures.

Each compression session involved a maximum of two participants, accompanied by an attendant. They were seated 1.5 m apart, with test screens positioned 1 m in front of them. Communication was maintained via headsets with microphones, and participants used wireless mice, mouse pads, and paper forms to complete tasks. A router placed outside the chamber ensured stable internet access.

### 2.3. Flicker Test

To assess CFFF, we used a device previously described [9]. It was a small handheld device equipped with a blue LED that flashed at frequencies ranging from 10 to 50 Hz, increasing at a rate of 1 Hz per second. Participants held the device approximately 30 cm from their eyes. Their task was to press a designated button at the moment they could no longer perceive the flickering of the light (fusion trial) and again when the flickering became visible once more (flicker trial). The device displayed three values: the frequencies recorded during the fusion and flicker trials, as well as the average of these two measurements. Participants noted their results on paper forms provided at the start of the session. All three values (fusion threshold, flicker threshold, and their average) were recorded once at each time point and were analyzed separately to capture potential differential sensitivity measurement protocols to hyperbaric conditions.

### 2.4. Cognitive Assessment

Cognitive tasks were delivered online using oil-immersed Alltab 4.0 tablets (Valtamer, Helsinki, Finland), certified for safe operation under hyperbaric conditions. Participants responded via wireless mice. All tasks were programmed in PsyToolkit software (version 3.4.6) [25,26]. The test battery was designed to assess selected cognitive functions within a short timeframe (10–15 min). Before participation, each individual was thoroughly briefed on the procedure and tasks. During each session, the same set of tests was administered, each preceded by a brief instruction. To minimize learning effects, task trials were randomly generated.

The cognitive test battery consisted of the Digit Span task, the Corsi Block-Tapping task (hereafter referred to as Corsi), and the Simon task. Both the Digit Span and Corsi Block-Tapping tasks assess the ability to store and reproduce sequences—verbal in the case of Digit Span, and visuospatial in the case of Corsi. Forward trials (reproducing sequences in the same order) primarily measure short-term memory and attention, while backward trials (reverse order) additionally engage working memory capacity [27,28,29,30]. Stimuli were presented at a rate of one per second.

In the Digit Span task, digits appeared sequentially on the screen, and participants reproduced the sequence using an on-screen keypad after the presentation ended. In the Corsi task, participants viewed a digital board with white squares arranged according to the original layout by Kessels et al. [30]. During each trial, individual squares lit up in green one at a time. After the sequence, participants used a mouse to click the squares in the correct order. The length of the presented sequences increased progressively from 2 to 9 elements, depending on the participant’s performance. If a participant provided two consecutive incorrect responses at the same sequence length, the task was terminated, and the length of the longest correctly reproduced sequence was recorded as the participant’s score.

The Simon task was used to assess inhibitory control and the ability to suppress cognitive interference from conflicting spatial cues [31,32]. Two white circles were displayed on the left and right sides of the screen. During each trial, one circle changed color to either green or red. Participants were instructed to respond based on color—left click for green, right click for red—regardless of the circle’s position. Performance was measured by reaction time (ms) and response accuracy.

### 2.5. Data Preparation

Before conducting the statistical analysis, the dataset was organized and screened. No individual outliers were excluded. From the Simon task, a total of 16,350 responses were collected (50 per participant in each condition). We removed 306 incorrect responses and 13 responses that exceeded the maximum response time (5 s), resulting in 16,031 valid responses (98.05%) for subsequent reaction time analyses. Due to the non-normal distribution of the data, median reaction times were calculated for each participant separately for congruent and incongruent trials, under three breathing conditions (air, heliox, trimix) and across three experimental phases (before, 4 ATA, after). In the datasets obtained from the memory tasks (Corsi Block-Tapping and Digit Span), five values equal to zero were identified. These were removed and treated as missing data as zero scores are extremely rare in healthy young adults. Normative studies report minimum scores of 2–3 for Corsi [30,33] and 4–5 for Digit Span [34,35] suggesting technical recording errors rather than true performance.

To allow for a direct comparison between the flicker test and the neuropsychological task results, raw scores were converted into relative percentage values. Median reaction times obtained during the initial stage of the experiment (before) served as the reference point, set at 100%. Reaction times recorded at subsequent stages (4 ATA and after) were expressed as percentage changes relative to this baseline. All computations were performed separately for each participant, each experimental stage, and each of the three breathing mixtures and administered tasks.

### 2.6. Statistical Analysis

Statistical analysis was conducted using IBM SPSS Statistics version 29.0.0.0 (IBM Corp., Armonk, NY, USA). The normality of all numerical variables was assessed using the Shapiro–Wilk test. The results indicated that the data deviated from a normal distribution; therefore, nonparametric tests were applied in subsequent analyses. Comparisons across multiple experimental conditions were performed using the Friedman test for repeated measures. Where appropriate, post hoc pairwise comparisons were carried out with the Wilcoxon signed-rank test. Differences between independent groups (based on participants’ sex) were examined using the Mann–Whitney U test, and associations between variables were assessed using Spearman’s rank correlation coefficient. Statistical significance was set at *p* < 0.05. Variations in sample sizes across analyses reflect incomplete participation across all three gas conditions and occasional missing trials; to retain as much data as possible for the analyses, pairwise deletion was applied. This approach allowed the inclusion of all available cases for each comparison, resulting in minor variations in sample size across analyses. The exact number of data points included in each analysis is reported alongside the corresponding results.

## 3. Results

A comparative analysis of the normalized results between women and men was conducted using the Mann–Whitney U test. Since the initial scores for each participant were set at 100%, they were excluded from the analysis; instead, we compared the normalized results for both sexes at the 4 ATA and after stages. The analysis revealed no statistically significant differences between women and men across any conditions and tests, as all *p*-values remained well above the predetermined significance threshold. Therefore, subsequent analyses combined both sexes. Descriptive statistics for the entire sample are presented in Table 1.

We also compared the normalized results of the flicker test and cognitive assessments obtained during exposure to different breathing gas mixtures at 4 ATA to evaluate the effects of these mixtures. The results—both for the flicker test and the neuropsychological assessments—obtained at 4 ATA and at the “after” stage did not differ significantly between the breathing mixtures (all *p*-values in Friedman tests were well above the significance threshold).

### 3.1. Relative Changes

**Figure 1:**
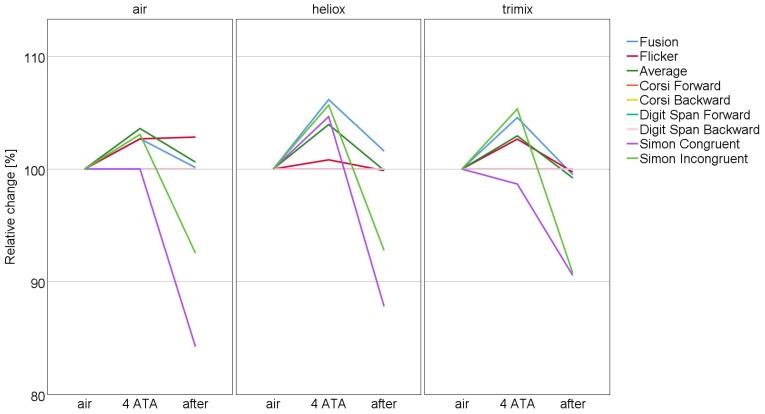
Median percentage changes in flicker test and neuropsychological test scores across breathing gas mixtures and study stages. Note: Lines for Digit Span Forward, Corsi Forward, and Corsi Backward overlap with the Digit Span Backward line at 100%, as median values remained unchanged from baseline across all time points.

For each breathing mixture, a separate Friedman test was performed on the flicker test and neuropsychological test results across the different stages of the study. Subsequently, pairwise comparisons between the study stages (before–4 ATA, 4 ATA–after, and before–after) were performed to identify the sources of the observed differences, or as an exploratory analysis in cases where no overall differences were detected. The results for each breathing mixture are described in the following sections, and the outcomes are also presented graphically in Figure 1.

#### 3.1.1. Flicker Test—Air

Fusion trial results showed significant variation between the study stages (N = 35, χ^2^ = 6.171, df = 2, *p* = 0.046). No statistically significant differences were obtained for flicker results (N = 35, χ^2^ = 5.314, df = 2, *p* = 0.070) and averaged results (N = 35, χ^2^ = 5.886, df = 2, *p* = 0.053), although *p*-values are very close to reaching the significance threshold. Pairwise comparisons revealed that results obtained at 4 ATA were significantly higher than initial values for flicker (Z = −2.352, *p* = 0.019) and average CFFF (Z = −2.433, *p* = 0.015), but not for fusion, where only a statistical trend was observed (Z = −1.922, *p* = 0.055). At the subsequent “after” stage, a significant decline was noted compared to the 4 ATA stage for fusion (Z = −2.944, *p* = 0.003) and average (Z = −2.540, *p* = 0.011), while flicker remained unchanged (Z = −0.632, *p* = 0.528). Additionally, final-stage values were comparable to initial stage results for fusion (Z = −0.148, *p* = 0.882) and average (Z = −0.484, *p* = 0.628), but not for flicker, which maintained its earlier increase (Z = −2.298, *p* = 0.022).

#### 3.1.2. Flicker Test—Heliox

Statistically significant differences between study stages were observed for fusion (N = 31, χ^2^ = 8.968, df = 2, *p* = 0.011) and average CFFF scores (N = 31, χ^2^ = 9.742, df = 2, *p* = 0.008). No significant differences were found for flicker scores (N = 31, χ^2^ = 3.355, df = 2, *p* = 0.187). However, pairwise comparisons revealed a significant increase at 4 ATA compared to baseline for all three measures: fusion (Z = −3.174, *p* = 0.002), flicker (Z = −2.058, *p* = 0.040), and average (Z = −3.503, *p* < 0.001). This was followed by a significant decrease in scores at the final stage: fusion (Z = −3.126, *p* = 0.002), flicker (Z = −2.357, *p* = 0.018), and average (Z = −3.865, *p* < 0.001). Final stage values returned to levels comparable to baseline for fusion (Z = −1.398, *p* = 0.162), flicker (Z = −0.990, *p* = 0.322), and average (Z = −0.016, *p* = 0.987).

#### 3.1.3. Flicker Test—Trimix

Significant differences across study stages were observed for all three trials: fusion (N = 28, χ^2^ = 9.071, df = 2, *p* = 0.011), flicker (N = 28, χ^2^ = 6.643, df = 2, *p* = 0.036), and average CFFF (N = 28, χ^2^ = 6.929, df = 2, *p* = 0.031). The pattern revealed by pairwise comparisons closely resembles that observed for heliox: post-decompression results are comparable to baseline values (fusion: Z = −0.460, *p* = 0.645; flicker: Z = −0.355, *p* = 0.723; average: Z = −0.988, *p* = 0.323), while results at 4 ATA are significantly increased compared to initial measurements (fusion: Z = −3.236, *p* = 0.001; flicker: Z = −2.844, *p* = 0.004; average: Z = −2.768, *p* = 0.006). Furthermore, when comparing the 4 ATA stage to the final stage, a significant decline was observed for fusion (Z = −3.990, *p* < 0.001) and average (Z = −3.613, *p* < 0.001), whereas flicker did not show a statistically significant change, although the result approached significance (Z = −1.901, *p* = 0.057). Also, as illustrated in Figure 1, the final flicker values closely resemble those recorded at baseline and align with fusion and average scores at this stage.

#### 3.1.4. Cognitive Assessment

Reaction times in the Simon task varied significantly across study stages for both congruent and incongruent trials under all breathing conditions. Specifically, significant differences were observed for congruent trials with air (N = 35, χ^2^ = 14.345, df = 2, *p* = 0.001), heliox (N = 31, χ^2^ = 16.710, *p* < 0.001), and trimix (N = 28, χ^2^ = 15.500, *p* < 0.001), as well as for incongruent trials with air (χ^2^ = 30.841, *p* < 0.001), heliox (χ^2^ = 23.290, *p* < 0.001), and trimix (χ^2^ = 19.838, *p* < 0.001). Figure 1 illustrates the percentage changes in reaction times across stages for both trials under each breathing condition. Pairwise comparisons showed no significant differences between baseline and 4 ATA for congruent trials (air: Z = −0.051, *p* = 0.960; heliox: Z = −1.047, *p* = 0.295; trimix: Z = −0.692, *p* = 0.489). For incongruent trials, no significant change was observed between baseline and 4 ATA in the air condition (Z = −0.596, *p* = 0.551), while significant differences were found for heliox (Z = −2.412, *p* = 0.016) and approached significance for trimix (Z = −1.945, *p* = 0.052). Highly significant differences were observed between baseline and after stages for both congruent (air: Z = −4.675, *p* < 0.001; heliox: Z = −2.985, *p* = 0.003; trimix: Z = −3.650, *p* < 0.001) and incongruent trials (air: Z = −4.200, *p* < 0.001; heliox: Z = −3.299, *p* < 0.001; trimix: Z = −3.154, *p* = 0.002). Similarly, comparisons between 4 ATA and after stages revealed significant decreases in reaction times for congruent trials (air: Z = −3.415, *p* < 0.001; heliox: Z = −2.973, *p* = 0.003; trimix: Z = −3.527, *p* < 0.001) and incongruent trials (air: Z = −4.271, *p* < 0.001; heliox: Z = −4.301, *p* < 0.001; trimix: Z = −3.342, *p* < 0.001). Although the difference between congruent and incongruent trials under heliox appears slightly larger than under air or trimix (see Figure 1), statistical analysis did not confirm a significant difference (N = 32, M_congruent_ = 102.637%, M_incongruent_ = 106.649%, Z = −1.085, *p* = 0.278).

No significant changes in memory test performance were observed across the different stages of the study. As shown in the descriptive statistics presented in Table 1, the variations in scores were minimal. Mean percentage changes remained within a few percent, and the medians were consistently equal to 100% across all conditions. However, the results exhibited considerable variability, as indicated by standard deviations.

### 3.2. Flicker Test and Neuropsychological Assessment

We conducted a correlation analysis between relative flicker test results and the outcomes of neuropsychological tests. The results of these analyses are presented in Table 2. Significant, albeit weak, correlations were found for incongruent trials in the Simon task with fusion (r = 0.110, *p* = 0.048) and average CFFF (r = 0.123, *p* = 0.028), but not with flicker (r = 0.049, *p* = 0.379). However, changes in flicker trials were significantly correlated with changes in Corsi forward scores (r = 0.120, *p* = 0.032) and digit span backward scores (r = 0.144, *p* = 0.010), although these correlations were also weak. In contrast, no significant correlations were observed between flicker results and Corsi backward (r = −0.022, *p* = 0.690) or digit span forward (r = 0.072, *p* = 0.200). No other subtests showed significant associations (see Table 2).

To examine whether relative changes in the flicker test differ from those observed in the neuropsychological assessments, we conducted a statistical analysis using the Wilcoxon Signed Ranks Test. The “before” stage was excluded from comparisons, as all values were equal to 100%. Analyses were performed separately for each breathing mixture. At 4 ATA, the relative changes in reaction times were comparable to those observed in the flicker test, with no *p*-values approaching the threshold for statistical significance. Similarly, no significant differences were found between changes in the flicker test and those in memory tasks (Corsi and digit span). In contrast, at the “after” stage, significant differences emerged between changes in flicker test results and reaction times changes in both congruent and incongruent trials of the Simon task (all *p* < 0.01), indicating a greater reduction in reaction times compared to flicker performance. For memory tests, no significant differences were observed at this stage either.

## 4. Discussion

The present study examined the association between CFFF and cognitive performance during exposure to hyperbaric conditions at 4 ATA while breathing different gas mixtures. Overall, our findings reveal a complex pattern of responses that varied across measurement domains, breathing gases, and recovery phases.

### 4.1. Critical Flicker Fusion Frequency Changes Due to Hyperbaric Exposure and Effects of Breathing Gas Composition

We found that CFFF values increased consistently at 4 ATA (approximately 3–5% above baseline) across all breathing gas mixtures, confirming that hyperbaric exposure alters neural temporal processing capacity. However, recovery patterns after decompression differed markedly between gases: flicker thresholds remained elevated following air breathing but returned to baseline after heliox and trimix.

This divergence likely reflects the distinct physicochemical properties of nitrogen and helium and their differing interactions with neural tissues [11,12]. Nitrogen’s high lipid solubility [36] allows it to dissolve readily into lipid-rich neuronal membranes, where it can alter membrane fluidity and modulate ion channel function and neurotransmitter receptor activity, according to the Meyer–Overton rule [37,38,39]. Because nitrogen washes out slowly from lipid-rich neural tissues [40,41] (due to the combination of high nitrogen’s solubility and relatively low blood perfusion), these alterations in neuronal excitability may persist after decompression. The persistence of elevated flicker thresholds specifically suggests that the neural processes underlying flicker perception may be particularly sensitive to these lingering nitrogen effects.

The different recovery patterns of fusion and flicker further support this interpretation. Fusion values returned to baseline, whereas flicker remained elevated, implying that these components rely on partly distinct neural mechanisms. Fusion may depend more on early sensory integration in subcortical structures, whereas flicker detection likely involves higher-level cortical processes that are more vulnerable to small, nitrogen-induced changes in membrane properties [42,43,44,45,46].

In contrast, helium’s minimal lipid solubility and rapid diffusion limit tissue accumulation, allowing immediate resolution of effects upon decompression. It is consistent with the full return to baseline after heliox breathing. Trimix, with only 36.7% nitrogen compared to 78% in air, showed the same recovery pattern as heliox, suggesting that sufficient helium content prevents the lingering neural alterations observed under air.

The absence of gas-specific differences at 4 ATA, despite subsequent divergent recovery, suggests that acute pressure-related effects (hyperoxia, mechanical compression) dominate during exposure itself, with gas-dependent differences emerging only during washout. The overall CFFF elevation at 4 ATA likely reflects altered neural excitability resulting from hyperoxic conditions induced by increased oxygen partial pressure.

#### Implications and Interpretation

These differential recovery patterns observed across breathing gases have several important implications. First, they suggest that the choice of breathing gas influences not only neural function during hyperbaric exposure but also the temporal profile of recovery after decompression. The persistence of elevated flicker thresholds after air breathing suggests that neural recovery may require 15–30 min beyond surfacing, even after physiologically safe decompression. This has practical relevance for post-dive activities requiring optimal sensory and cognitive performance, particularly in multi-dive operations where rapid neural recovery is desirable. While helium-based gases showed faster recovery, cost considerations limit their use in recreational diving, though they may benefit professional operations requiring rapid return to duty. This finding aligns with Balestra et al.’s [3] observation of prolonged neural effects following air diving (33 msw, 20 min), though the direction of change differed (elevation in our chamber exposures vs. impairment in their wet dives). Despite methodological differences, both studies converge on the key finding that neural alterations persist post-decompression when breathing air, supporting the need for post-dive cognitive recovery intervals. Also, the magnitude of these CFFF changes (3–5% at 4 ATA) warrants careful interpretation. While Muth et al. [47] suggested that interventions should exceed approximately 4% variability observed in a single individual, the literature consistently reports percentage changes in CFFF of similar magnitude [47]. Our results align with Balestra et al. [3] at identical pressure and with other studies demonstrating that a few percent difference represents the typical response range in this field, particularly when systematic gas-dependent patterns and correlations with cognitive performance provide convergent validity.

Second, the finding that fusion and average CFFF components normalized while flicker remained elevated suggests that different CFFF measures may capture distinct aspects of visual system function with different sensitivities to residual gas effects, highlighting the value of analyzing components separately.

Third, the complete normalization of all CFFF components after heliox and trimix breathing supports the use of helium-based mixtures when rapid neural recovery is desired. The faster washout of helium may provide advantages in contexts requiring multiple exposures or quick recovery.

Finally, the overall elevation of CFFF at 4 ATA, regardless of breathing mixture, likely reflects altered neural excitability from the hyperoxic environment created by increased ambient pressure. This aligns with Kot et al.’s findings [10] that hyperoxia’s effect on neuronal excitability is dose-dependent, with higher oxygen pressures (2.8 ATA) increasing neural activity as reflected by elevated CFFF. This hyperoxia-related modulation appears consistent across all our breathing conditions, while post-decompression persistence is determined primarily by inert gas elimination kinetics.

To better understand the specific contribution of inert gases beyond the hyperoxia-related effects observed at 4 ATA, we decided to examine air, heliox, and trimix rather than nitrox. Nitrox would have introduced confounding hyperoxia at 4 ATA (pO_2_ 1.3–1.5 ATA), complicating interpretation since oxygen effects on CFFF have been characterized previously [9,10,48]. By maintaining similar inspired oxygen fractions across all mixtures while maximizing nitrogen contrast (78% in air, 0% in heliox, 36.7% in trimix), we could attribute gas-dependent recovery patterns specifically to nitrogen elimination kinetics rather than oxygen effects.

### 4.2. Relationship Between Critical Flicker Fusion Frequency and Cognitive Performance

The relationship between CFFF and cognitive performance revealed a theoretically informative pattern. As hypothesized, hyperbaric stress revealed selective CFFF–cognition associations absent under normobaric conditions [7], confirming that arousal mechanisms become functionally coupled when homeostatic systems are challenged [1,4]. This supports CFFF’s utility as an operational readiness indicator, particularly for prefrontal-dependent executive tasks.

The Simon task and CFFF measures revealed complementary yet distinct patterns of neural and cognitive responses during hyperbaric exposure. At 4 ATA, both reaction times and CFFF values exhibited modest increases (3–5%), likely reflecting physiological factors, such as altered neural excitability [10] or transient modulation of sensory processing, were influencing performance. While CFFF, a measure primarily sensitive to temporal resolution in the visual system, returned to baseline after decompression, reaction times continued to improve substantially. This divergence highlights the different mechanisms: CFFF reflects immediate changes in sensory processing that normalize as environmental stress subsides, whereas the Simon task, involving stimulus discrimination, response selection, and motor execution, might be sensitive to accumulated practice and cognitive adaptation [49,50].

Correlation analyses revealed that fusion and average CFFF showed weak but significant associations with incongruent (conflict-demanding) Simon task performance, while flicker correlated slightly with memory-related tasks. These patterns indicate that CFFF and traditional neuropsychological measures capture overlapping but largely distinct aspects of neural functioning, with fusion reflecting lower-level sensory processing, and flicker showing sensitivity to cortical mechanisms linked to memory and higher-order cognitive integration. This multifaceted nature of flicker processing was described previously in our review [51].

Taken together, these findings paint a nuanced picture of neural adaptation under hyperbaric conditions. Simple perceptual measures like CFFF provide insight into immediate, physiologically driven changes in neural excitability that continue to evolve even after the stressor is removed. The emergence of selective CFFF–cognition correlations under hyperbaric stress—where such correlations were absent under normobaric conditions—suggests that shared arousal mechanisms become functionally coupled when homeostatic systems are challenged. This complementary relationship underscores the value of incorporating both types of measures in operational environments: CFFF offers a rapid, learning-resistant assessment of instantaneous arousal state, while task-based cognitive measures capture performance capacities in specific functional domains. The finding that these measures become associated specifically under stress conditions has important implications for using CFFF as a practical monitoring tool in extreme environments, where it may detect arousal perturbations that translate into cognitive performance changes.

### 4.3. Sex Differences: Absence of Effects

Our analysis revealed no significant differences between women and men in any of the measured outcomes. This finding is consistent with some previous research suggesting that basic cognitive functions and neural processing capabilities do not differ substantially between sexes under standard testing conditions [6,52,53,54,55,56,57,58,59]. While some studies report sex differences in CFFF [60,61], reaction time [62,63,64], and memory tasks [65,66], these effects are typically small and observed primarily in very large groups [67], task-dependent [66] or connected with other factors [56,68,69].

The absence of sex differences observed here is practically beneficial for diving, suggesting that men and women maintain similar cognitive and neural functioning under these hyperbaric conditions. This may reflect the characteristics of our young, healthy samples, the stringent inclusion criteria that selected physiologically similar individuals, and the possibility that the experimental conditions were not challenging enough to reveal sex-specific stress responses. Further research with larger, more diverse samples and more demanding exposures is needed to confirm this equivalence.

### 4.4. Potential Involvement of the Noradrenergic System and Arousal Dynamics

While we did not directly measure LC activity or arousal dynamics, several features of our results align with predictions from arousal-related frameworks, outlined in the introduction. First, the parallel modest increases in both CFFF values and reaction times at 4 ATA could reflect a common arousal state shift in response to hyperbaric stress. The LC is exquisitely sensitive to physiological perturbations including hyperoxia, hypercapnia, and metabolic challenge [70], which are features of hyperbaric exposure. Increased LC firing elevates cortical norepinephrine, potentially modulating neural excitability in thalamocortical circuits [16,18] determining CFFF thresholds. If this interpretation holds, the CFFF increase at depth may serve as an indirect marker of heightened central arousal.

Second, the selective correlations between CFFF and conflict-demanding Simon task trials (but not congruent trials or memory tasks) align with PFC sensitivity to noradrenergic modulation [16,19]. The absence of correlations with memory tasks reflects that the differential dependence of PFC executive systems on α2A-mediated LC–NE modulation [19,20,21] versus medial temporal lobe β-adrenergic mechanisms f [22,23].

Third, the remarkable post-decompression improvement in reaction times (10–15% faster than baseline) could reflect complex interactions between arousal normalization, practice effects, and PFC optimization. Upon return to surface pressure, the removal of hyperbaric stressors may have allowed PFC circuits to operate within their optimal noradrenergic range, while benefiting from task familiarity. The selectivity of this improvement for the Simon task but not memory tasks supports the hypothesis that PFC-dependent processes were particularly affected by arousal perturbations during hyperbaric exposure.

Perhaps most intriguingly, the differential CFFF recovery patterns across breathing gases also invite consideration of arousal-related mechanisms. The persistence of elevated flicker thresholds after air breathing but not after heliox or trimix is difficult to explain through purely sensory or metabolic mechanisms. Thus, nitrogen’s slow washout from lipid-rich neural membranes may subtly modulate LC neuronal firing or the responsiveness of LC target regions to noradrenergic input, maintaining elevated temporal processing capacity even after pressure normalizes.

The selectivity of this effect for flicker detection, rather than fusion or average CFFF, might reflect differential sensitivity of the neural circuits underlying temporal breakdown detection to sustained membrane alterations. Alternatively, nitrogen’s lingering presence could directly influence thalamocortical gain control mechanisms independent of arousal, or the observed pattern could simply reflect statistical noise given our modest sample sizes for individual gas conditions.

However, these LC-related interpretations remain speculative. Our study lacked direct measures of arousal (pupillometry, heart rate variability), sympathetic activity, or LC function. The observed correlations, while theoretically meaningful, are weak and could arise from alternative mechanisms including cerebrovascular effects, metabolic changes [71], or breathing mechanics. Nevertheless, the convergence of parallel CFFF and executive function changes at depth, selective correlations with conflict-demanding cognition, gas-dependent persistence of effects, and theoretical plausibility of LC involvement suggest that arousal-related mechanisms merit consideration in future research. Notably, recent theoretical work [72] proposing that various physiological and behavioral measures represent distinct nonlinear projections from multidimensional LC–NE arousal dynamics provides a framework that could potentially unify our disparate findings, though direct empirical testing in hyperbaric contexts remains to be conducted.

### 4.5. Limitations

Several limitations of this study should be acknowledged. First, the relatively brief exposure duration (about 30 min) may have been insufficient to detect certain effects that would emerge during longer exposures. Second, the testing battery, while comprehensive, was necessarily brief to be feasible under hyperbaric conditions and may have lacked sensitivity to subtle cognitive changes. Third, the sample consisted of healthy young adults, limiting generalizability to other populations such as older individuals or those with pre-existing conditions. Fourth, our correlational findings should be interpreted cautiously as weak correlations could result from numerous factors beyond shared arousal modulation, including individual differences in test-taking strategies or motivation.

### 4.6. Future Directions and Clinical Applications

Despite some limitations, our findings suggest several promising directions for future research that could more directly test the hypotheses we have proposed. Future research should directly assess LC and arousal dynamics using ultra-high field MRI to link LC integrity and activity with individual variability in CFFF modulation. High-density EEG combined with pupillometry could reveal how network activity changes across flicker frequencies and arousal levels, enabling direct testing of arousal-linked modulation of temporal visual processing. Integrated multimodal studies and computational modeling could help specify how noradrenergic modulation determines individual CFFF thresholds. Until such studies are conducted, our arousal-related interpretation should be viewed as a plausible but unconfirmed hypothesis.

Better understanding of CFFF’s relationship to arousal could have practical value in operational settings such as diving, aviation, and spaceflight, helping optimize performance under physiological stress. Clinically, altered CFFF responses could provide information about disorders involving LC degeneration (Parkinson’s, Alzheimer’s), attention regulation (ADHD), or arousal control (narcolepsy, insomnia). If validated, CFFF could serve as a simple, non-invasive biomarker for monitoring disease progression and treatment effects.

## 5. Conclusions

This study demonstrates that acute hyperbaric exposure at 4 ATA produces measurable CFFF increases with gas-dependent recovery dynamics: flicker thresholds remained elevated after air breathing but normalized rapidly after heliox and trimix, indicating that nitrogen’s slow tissue washout prolongs neural effects beyond the hyperbaric period with implications for post-dive cognitive readiness. The weak correlations between CFFF and some cognitive measures indicate that these assessment tools capture somewhat different aspects of neural functioning and may serve complementary roles in evaluating neurocognitive status during hyperbaric exposure. These findings suggest that surface interval recommendations should consider cognitive recovery alongside decompression safety, and that nitrogen-free mixtures offer protective advantages even at recreational depths. Future research should validate CFFF as a practical readiness indicator and test the hypothesized LC-mediated mechanisms through direct arousal measurements, neurochemical assays, and neuroimaging approaches.

## Figures and Tables

**Table 1:** Descriptive statistics of normalized scores obtained in the flicker test (fusion, flicker, average) and neuropsychological assessments across different study stages.

Test	Stage	N	M	Me	SD
Fusion	4 ATA	112	104.57	104.35	9.69
	after	113	100.36	100.15	7.38
Flicker	4 ATA	112	102.95	101.57	7.30
	after	113	100.70	100.20	6.67
Average	4 ATA	112	103.41	103.60	6.72
	after	113	100.22	99.95	5.34
Corsi Forward	4 ATA	102	99.13	100.00	27.39
	after	110	103.64	100.00	27.44
Corsi Backward	4 ATA	102	103.92	100.00	37.29
	after	108	105.10	100.00	40.01
Digit Span Forward	4 ATA	102	100.80	100.00	20.28
	after	110	104.59	100.00	19.17
Digit Span Backward	4 ATA	101	108.38	100.00	28.81
	after	110	109.54	100.00	33.86
Simon Congruent	4 ATA	103	102.66	101.01	22.17
	after	109	87.31	87.63	17.53
Simon Incongruent	4 ATA	103	105.02	104.44	16.45
	after	109	91.00	92.49	16.87

N = number of observations; M = mean; Me = median; SD = standard deviation. Results are presented for the study stages “4 ATA” and “after.” The “before” stage was excluded, as all values were identical and equal to 100%.

**Table 2:** Spearman correlations between percentage changes in flicker test scores and neuropsychological test results.

Test	Fusion			Flicker			Average		
	N	r	*p*	N	r	*p*	N	r	*p*
Simon Congruent	322	0.063	0.261	322	−0.083	0.138	322	0.051	0.362
Simon Incongruent	322	0.110	0.048 *	322	0.049	0.379	322	0.123	0.028 *
Corsi Forward	321	−0.055	0.328	321	0.120	0.032 *	321	0.025	0.653
Corsi Backward	317	−0.093	0.098	317	−0.022	0.690	317	−0.057	0.315
Digit Span Forward	321	−0.038	0.495	321	0.072	0.200	321	0.011	0.841
Digit Span Backward	320	0.003	0.953	320	0.144	0.010 *	320	0.013	0.822

N = number of observations; r = Spearman’s rank correlation coefficient; *p* = statistical significance level; * indicates statistically significant correlations (*p* < 0.05).

## Data Availability

The data presented in this study are available on request from the corresponding author.
